# Chemical Composition, Biological Activity, and Application of *Rosa damascena* Essential Oil as an Antimicrobial Agent in Minimally Processed Eggplant Inoculated with *Salmonella enterica*

**DOI:** 10.3390/foods13223579

**Published:** 2024-11-09

**Authors:** Andrea Verešová, Milena D. Vukic, Nenad L. Vukovic, Margarita Terentjeva, Zhaojun Ban, Li Li, Alessandro Bianchi, Ján Kollár, Rania Ben Saad, Anis Ben Hsouna, Joel Horacio Elizondo-Luévano, Maciej Ireneusz Kluz, Natália Čmiková, Stefania Garzoli, Miroslava Kačániová

**Affiliations:** 1 Institute of Horticulture, Faculty of Horticulture and Landscape Engineering, Slovak University of Agriculture, Tr. A. Hlinku 2, 94976 Nitra, Slovakia; andrea.veresova1979@gmail.com (A.V.); n.cmikova@gmail.com (N.Č.); 2 Department of Chemistry, Faculty of Science, University of Kragujevac, 34000 Kragujevac, Serbia; milena.vukic@pmf.kg.ac.rs (M.D.V.); nvchem@yahoo.com (N.L.V.); 3 Faculty of Veterinary Medicine, Latvia University of Life Sciences and Technologies, LV-3001 Jelgava, Latvia; margarita.terentjeva@llu.lv; 4 Zhejiang Provincial Key Laboratory of Chemical and Biological Processing Technology of Farm Products, Zhejiang Provincial Collaborative Innovation Center of Agricultural Biological Resources Biochemical Manufacturing, School of Biological and Chemical Engineering, Zhejiang University of Science and Technology, Hangzhou 310023, China; banzhaojun@zust.edu.cn; 5 Key Laboratory for Agro-Products Postharvest Handling of Ministry of Agriculture and Rural Affairs, College of Biosystems Engineering and Food Science, Zhejiang University, Hangzhou 310058, China; lili1984@zju.edu.cn; 6 Department of Agriculture, Food and Environment, University of Pisa, Via del Borghetto 80, 56124 Pisa, Italy; alessandro.bianchi@phd.unipi.it; 7 Institute of Landscape Architecture, Faculty of Horticulture and Landscape Engineering, Slovak University of Agriculture, Tulipánová 7, 94976 Nitra, Slovakia; jan.kollar@uniag.sk; 8 Laboratory of Biotechnology and Plant Improvement, Centre of Biotechnology of Sfax, B.P “1177”, Sfax 3018, Tunisia; raniabensaad@gmail.com (R.B.S.); benhsounanis@gmail.com (A.B.H.); 9 Department of Environmental Sciences and Nutrition, Higher Institute of Applied Sciences and Technology of Mahdia, University of Monastir, Monastir 5000, Tunisia; 10 Department of Chemistry, Faculty of Biological Sciences, Universidad Autónoma de Nuevo León, San Nicolás de los Garza C.P. 64455, Nuevo León, Mexico; joel.elizondolv@uanl.edu.mx; 11 School of Medical & Health Sciences, University of Economics and Human Sciences in Warsaw, Okopowa 59, 01-043 Warszawa, Poland; m.kluz@vizja.pl; 12 Department of Chemistry and Technologies of Drug, Sapienza University, P. le Aldo Moro, 5, 00185 Rome, Italy; stefania.garzoli@uniroma1.it

**Keywords:** phytochemicals, food spoilage microorganisms, rose absolute, antimicrobial activity, insecticidal activity, food model

## Abstract

*Rosa damascena* is mostly grown for its usage in the food, medical, and perfume industries, while it is also used as an attractive plant in parks, gardens, and homes. The use of *R. damascena* essential oil may yield new results in relation to the antimicrobial activity of essential oils and their use mainly in extending the shelf life of foods. This study investigates the chemical composition and antimicrobial properties of *Rosa damascena* essential oil (RDEO) using gas chromatography–mass spectrometry (GC-MS) and various bioassays to explore its potential applications in food preservation and microorganism growth control. The GC-MS analysis revealed that RDEO is predominantly composed of phenylethyl alcohol (70%), which is known for its antimicrobial and aromatic properties. Additionally, other significant constituents were identified, including nerol, citronellol, and geraniol, which may contribute to the EOs overall bioactivity. The antimicrobial activity was assessed through the minimal inhibition concentration against five *Candida* yeast strains, four Gram-positive, and four Gram-negative bacteria, including biofilm-forming *Salmonella enterica*. Determination of minimum inhibitory concentrations (MIC) revealed the strongest effects of RDEO’s on Gram-negative species, with MIC_50_ values as low as 0.250 mg/mL for *S. enterica*. Moreover, an in situ assessment utilizing fruit and vegetable models demonstrated that the vapor phase of RDEO significantly suppressed microbial growth, with the most substantial reductions observed on kiwi and banana models. As a result of our study, the antimicrobial effect of RDEO on the microbiota of sous vide processed eggplant was detected, as well as an inhibitory effect on *S. enterica* during storage. The insecticidal activity against *Megabruchidius dorsalis* Fahreus, 1839, was also studied in this work and the best insecticidal activity was found at the highest concentrations. These results suggest that RDEO has the potential to serve as a natural antimicrobial agent in food preservation and safety applications, providing an alternative to synthetic preservatives.

## 1. Introduction

A growing amount of produce purchased by consumers is minimally processed, especially organically produced fruits and vegetables [1]. Consumers are increasingly choosing convenient, ready-to-use produce that maintains fresh quality and contains only natural ingredients [2,3]. To extend the shelf life of fresh fruits and vegetables, the growth of microbial populations must be controlled [4]. Several postharvest procedures, such as washing and removing damaged tissues, are employed to reduce initial high microbial counts. However, this processing can negatively impact the plant tissues’ metabolism and may reduce the shelf life of the fruits or vegetables [5].

Fruits and vegetables have a short shelf life due to weight loss and decay, primarily caused by fungal activity. However, they are becoming increasingly recognized for their health benefits due to their nutrient and fiber content. To extend the shelf life of fruits and vegetables, essential oils (EOs) can be incorporated into protective biofilms or applied in modified atmosphere packaging (MAP) [6]. To offer consumers fresh produce more efficiently, the food industry introduced the concept of “fresh-cut food”, which refers to produce that has been physically altered but remains fresh. Fresh-cut food is ready to eat and appealing due to its fresh-like flavor, appearance, and taste [7]. It also offers health benefits, requiring less preparation time and effort. Since these products are pre-cleaned and cut, consumers save time in preparation. Fresh-cut fruits and vegetables (FCFV) are more convenient and easier to transport and consume than whole produce. However, there are significant challenges in maintaining their quality, freshness, and microbiological safety [5]. Although the studies show that the consumers are opting for fresh-cut products due to their health benefits, there has also been an increase in foodborne illnesses caused by bacteria such as *Salmonella enterica* (ex. Kauffmann and Edwards 1952), *Escherichia coli* (Migula 1895), *Shigella* spp., *Campylobacter* spp., *Listeria monocytogenes* (Murray et al. 1926), *Staphylococcus aureus* Rosenbach 1884, *Yersinia* spp., and *Bacillus cereus* Frankland and Frankland 1887 [8].

The use of the EOs as potent antimicrobial agents in minimally processed foods has been expected to exhibit a significant effect on safety of fresh produce and, hence, has attracted the attention of research [9]. Low storage temperatures and decreased pH enhance the antibacterial activity of EOs in fruit and vegetable-based foods [9]. Researchers have employed EOs in various studies to enhance the antimicrobial properties of fruits and vegetables, showing promising results [10,11,12,13,14,15]. The strong antibacterial activity of *Rosa damascena* EO and absolute was demonstrated by Ulusoy et al. [16] against strains of *Pectobacterium carotovorum* (Jones 1901), *Pseudomonas aeruginosa* (Schroeter 1872), *Bacillus subtilis* (Ehrenberg 1835), *Staphylococcus aureus* Rosenbach 1884, and *Chromobacterium violaceum* Bergonzini 1880. *Chromobacterium violaceum* was found to be the most susceptible to rose absolute and EO, while *E. coli* also showed sensitivity to rose EO. However, none of the microorganisms were affected by the antibacterial properties of the hydrosol [16]. Both Gram-positive and Gram-negative bacteria were also susceptible to the antibacterial properties of rose absolute [16].

*Rosa damascena* Mill., commonly known as the Damask rose, is a fragrant shrub belonging to the *Rosa* genus. It has over 18,000 varieties and approximately 200 species. Most roses are shrubs, found across the Northern Hemisphere’s temperate and subtropical zones [17,18]. On an industrial scale, *R. centifolia* L., *R. gallica* L., and *R. damascena* Mill. are primarily used for their fragrance and flavor. Among these, *R. damascena* is regarded as the highest quality EO producer [19]. While primarily cultivated in Bulgaria, Turkey, and Iran, this plant is also grown in China, India, Libya, Morocco, South Italy, South France, South Russia, and Ukraine [20,21]. The main products derived from *R. damascena* include rose water (hydrosol), essential oil, absolute, and concrete. The use of rose EO dates back to ancient Persia. The practice of distilling roses for their EO likely began in Persia during the late seventh century A.D. and spread to Ottoman territories [22,23]. As one or more synergists can provide the desired fragrance and flavor without adversely affecting the food, the use of EOs in the food industry and consumer goods is expected to increase in the future [24]. *Rosa damascena* is being used more and more in laboratories and industries to obtain essential oil because of its high product yield, low organic solvents, quick processing time, and cheap maintenance expenses [25].

The prevalence of *Salmonella* spp. in plants poses a significant contamination risk in the food industry. Additionally, *Salmonella* strains can form biofilms on various food contact surfaces, such as processing equipment, knives and cutting boards, leading to cross-contamination of vegetables [26,27]. Cleaning and sanitation practices, often involving chemical disinfectants like chlorinated water [28,29], are fundamental in reducing *Salmonella* infections. However, the use of chemical disinfectants raises concerns about sustainability and safety. Therefore, it is essential to develop “green washing solutions” as effective alternatives to chemical disinfectants [30].

It is well known that the bacterial growth phase can influence their sensitivity to antimicrobial treatments [31]. Therefore, the effectiveness of antibacterial agents could be affected by bacterial cell physiology. Understanding whether bacterial cells in different growth phases are more susceptible or resistant to treatments is crucial [32].

EO, their distillation co-products (hydrosols), and plant extracts have been studied for their potential use in the food industry. These natural alternatives have demonstrated antimicrobial, antioxidant, and food preservation properties [33,34]. Some EOs have even shown a potential in reducing *Salmonella* spp. on fruits and vegetables [35,36,37,38]. Biofilm formation occurs when bacteria adhere to surfaces, such as food processing equipment, and secrete extracellular polymeric substances, providing structural protection to bacterial communities [39]. Bacteria in biofilms are more resistant to the human immune system, fungicides, antibiotics, and environmental stress than planktonic bacteria [40,41]. Organic matter left after food processing creates favorable conditions for biofilm formation, which poses cross-contamination risks [42,43]. In fact, bacterial adhesion and biofilm formation on processing equipment surfaces were identified as primary contributors to cross-contamination in trace analyses of *Salmonella* contamination [44,45].

Insect control in post-harvest crops and stored grains has also received significant attention. Various methods are used to protect crops from pests after harvest [46]. Chemical pesticides are often employed in agricultural fields and storage facilities to prevent and control pests. However, excessive use of chemical pesticides can result in resistance, environmental harm, and toxicity to non-target species [47]. Plant-derived bioinsecticides, such as EOs, may offer a safe and effective alternative to chemical pesticides [48]. Numerous studies have demonstrated the potential of plant-derived compounds, including EOs, in the management of pets in stored grain [49,50].

Inspired by the positive biological features of *Rosa damascena* Mill. and its metabolites, this work provides an extensive analysis of the chemical composition and biological profile of *R. damascena* EO derived from fresh flowers (RDEO). The therapeutic benefits of *R. damascena* motivated a comprehensive study of RDEO’s bioactive effects, focusing on its antibacterial (in vitro and in situ), antibiofilm, and insecticidal properties. This study also explores the potential use of RDEO as a green storage protectant, specifically for handling preserved foods like sous vide eggplant contaminated with *Salmonella enterica*.

## 2. Materials and Methods

### 2.1. Essential Oil

The absolute 100% *Rosa damascena* Mill. essential oil (RDEO) used in this study was purchased from Hanus s.r.o. (Nitra, Slovakia). *R. damascena* was cultivated in Turkey. The first step in the process was the production of rose concretion, obtained by extracting the flowers of *R. damascena*. This fragrant waxy substance was then dissolved in pure ethanol and cooled. The frozen wax was filtered off, and the ethanol was fully evaporated from the solution, resulting in rose absolute.

### 2.2. GC/MS Examination of the Volatile Components in RDEO

The analysis on the volatile content of the *Rosa damascena* essential oil (RDEO) sample was conducted using an Agilent Technologies (Santa Clara, CA, USA) 6890N gas chromatograph. Prior to analysis, the EO was dissolved in a 10% hexane solution. The oven was set to operate at 50 °C and increased by 4 °C/min to 70 °C (holding for 2 min), then increased by 5 °C/min to 120 °C (holding for 1 min), and finally increased by 5 °C/min to 290 °C. The entire run took 52 min, with a 1 mL injection volume. Data collection was set to start after a 3.2 min solvent delay. Additionally, the MS quadrupole and MS ion source temperatures were 150 °C and 230 °C, respectively. The investigated sample was injected in split mode with a split ratio of 40.8:1. The carrier gas, helium 5.0, was used and flowed at a rate of 1 µL/min. Data were gathered in scan mode using electron impact mass spectrometry (EI-MS; 70 eV) in the 35–550 m/z range. To identify volatile compounds, retention indices were established experimentally using n-alkanes (C_7_–C_35_). These indices were then compared with those available in the NIST and Wiley databases. Compound percentages (amounts >0.1%) were calculated from their GC peak regions [51,52].

### 2.3. Microorganisms Tested for Antimicrobial Activity

The assessed EO antibacterial activity was evaluated using the following strains of bacteria: *Bacillus cereus* CCM 7934, *Listeria monocytogenes* CCM 4699, *Staphylococcus aureus* subsp. *aureus* CCM 4423, and *Streptococcus pneumoniae* CCM 4501, which are examples of Gram-positive bacteria; *Salmonella enterica* subsp. *enterica* CCM 3807, *Serratia marcescens* CCM 8588, *Shigella sonnei* CCM 4421, *Yersinia enterocolitica* CCM 7204T, which are examples of Gram-negative bacteria; and yeasts including *Candida albicans* CCM 8186, *Candida glabrata* CCM 8270, *Candida krusei* CCM 8271, *Candida parapsilosis* CCM 8260, and *Candida tropicalis* CCM 8223. For this experiment, all Gram-positive and Gram-negative bacterial species and yeasts were obtained from the Czech Collection of Microorganisms (CCM), which is kept in Brno, Czech Republic. From milk production, the biofilm-forming Gram-negative *Salmonella enterica* was isolated and sequenced to evaluate the antibacterial and antibiofilm activity. Before analysis, the bacterial and yeast inoculums were cultivated for 24 h at 37 °C and 25 °C, respectively, in Mueller–Hinton Broth (MHB, Oxoid, Basingstoke, UK) and Sabouraud Dextrose Broth (SDB, Oxoid, Basingstoke, UK). On the day of the experiment, the optical density of the bacterial and yeast inoculum was set at the 0.5 McFarland standard [53].

### 2.4. Minimal Inhibition Concentration (MIC)

The minimal inhibitory concentration values (MIC_50_ and MIC_90_) were calculated. The microbial inoculum was initially added to 50 μL of a 96-well microtiter plate. Different concentrations of RDEO [54] (10 mg/mL to 0.00488 mg/mL in MHB) were then added. The negative and positive controls were prepared using MHB and SDB with EO (at the appropriate concentration) and MHB and SDB with inoculum, respectively. After the incubation period, absorbance at 570 nm was measured using a spectrophotometer (Glomax, Promega Inc., Madison, WI, USA). The MIC_50_ and MIC_90_ values correspond to the lowest concentrations of RDEO that inhibit 50% and 90% of bacterial growth, respectively. The test was performed in triplicate.

### 2.5. Examination of the Fruit and Vegetables In Situ

To evaluate the antimicrobial properties of RDEO in situ, a variety of commercial kiwi, banana, eggplant, and pumpkin substrates were used, along with specific strains of yeast and Gram-positive and Gram-negative bacteria [55]. The substrates were chopped into 0.5 mm pieces, cleaned, and then placed into 60 mm Petri plates containing the bacteria. RDEO samples at concentrations of 500, 250, 125, and 62.5 μg/L were dispersed using ethyl acetate. Filter sheets made of ethyl acetate were used as controls. The Petri plates were sealed and incubated for seven days at 37 °C. The volume density of the bacteria and yeasts was calculated using the ImageJ program, and conventional techniques were employed to measure the in situ growth of microbial colonies [54].

### 2.6. Assay for Antibiofilm

#### 2.6.1. Crystal Violet Assay

In the crystal violet study, Kačániová et al. [56] incubated bacterial suspensions in Mueller–Hinton Broth at 37 °C to determine the minimum biofilm inhibitory concentration (MBIC). A microtiter plate containing an inoculum was treated with two-fold dilutions of EO (from 100 mg/mL to 0.049 mg/mL). Wells were cleaned, stained, and their absorbance at 570 nm was measured after a 24 h period. The doses that inhibit 100%, 50%, and 90% of biofilm formation were determined to be MBIC_50_ and MBIC_90_, respectively.

#### 2.6.2. Applying the MALDI-TOF MS Biotyper to Detect the Development of Biofilms

A Bruker Daltonics MALDI-TOF MicroFlex (Bruker Daltonics GmbH & Co. KG, Bremen, Germany) instrument was used to measure the protein degradation that occurs during the biofilm-forming process. Stainless steel and glass slides were placed into 50 mL polypropylene tubes containing 20 mL of Mueller–Hinton Broth (MHB) and 100 μL of *Salmonella enterica* biofilm-forming bacterial inoculum. Experimental tubes were filled with EO to a concentration of 0.1%, while control tubes were left undisturbed. Biofilms were removed from glass and plastic surfaces by shaking at 170× *g* for three days and then incubated at 37 °C for five, seven, nine, twelve, and fourteen days. Additionally, planktonic cells from control samples used for the RDEO experiment were investigated. Dendrograms based on the estimation of Euclidean distance using 19 typical global spectra were produced and protein spectra were obtained using MALDI-TOF in the linear positive mode [56].

### 2.7. Kinetics Growth Measurement

To develop growth curves for *Salmonella enterica*, optical density (OD) at 850 nm was measured using a personal bioreactor (RTS-1, Biosan, Riga, Latvia). The strain was first cultured for 24 h on Mueller–Hinton Agar (MHA; Oxoid, Basingstoke, UK) at 37 °C. One colony was then transferred to a 30 mL sealed tube containing Mueller–Hinton Broth (MHB) and incubated at 37 °C until the OD850 reached 1, representing the log phase of bacterial growth. The bioreactor operated at 2000 rpm with directional changes every second. The temperature was gradually raised to 50 °C, with OD readings recorded every 5 min over a 20 min period. This procedure was then repeated at 55 °C, 60 °C, and 65 °C. In a separate experiment, 1% EO was introduced to the culture at the point when OD850 reached 1, marking the log phase. OD readings were recorded for both control (no EO) and experimental (with EO) conditions. Notably, the bioreactor is calibrated for microorganisms of 0.4–0.8 × 1–3 μm in size.

### 2.8. Antimicrobial Action of Sous Vide in the Eggplant Model

The two and a half kg of eggplant samples used in this investigation came from an authorized dealer in the Slovak Republic. Utilizing a sterile knife, the eggplant (*Solanum melongena*) was cut into five-gram halves after cooling and was then transported to the microbiological laboratory. The study included a total of 480 five-gram samples: 3 raw samples, 240 treated and control samples on day 1, and 240 treated and control samples on day 7. The chopped eggplant samples were treated with a 1% *v*/*w* RDEO solution dissolved in rapeseed oil, and each was vacuum packed separately with a Concept vacuum packer. Both vacuum-packed and unpackaged samples were employed as control samples. Samples containing 100 µL of *S. enterica* and 1% *v*/*w* RDEO were intended to imitate the presence of *S. enterica* without harming the eggplant. Before the samples were vacuum packed, they were stimulated for approximately a min [57].

We had the following data accessible to us while we were assessing it:

(i) Control: New eggplant samples were kept at 4 °C in polyethylene bags. Following this, they underwent treatments at 50 to 65 °C for 5 to 20 min.
(ii) Control + vacuum: Fresh eggplant samples were treated for 5 to 25 min at 50–65 °C after being vacuum packed in polyethylene bags and kept at 4 °C.
(iii) EO: Collected eggplant samples received vacuum packaging, 1% RDEO treatment, and preservation at 4 °C. After that, they were cooked for 5 to 25 min at 50 to 65 °C.
(iv) *Salmonella*: Fresh eggplant samples that were vacuum packed and treated with *S. enterica* were kept at 4 °C before being exposed to the bacterium for 5 to 25 min at 50 to 65 °C.
(v) *Salmonella* + EO: Vacuum-packed fresh eggplant samples treated with *S. enterica* and containing 1% RDEO were held at 4 °C before being treated for 5 to 25 min at 50 to 65 °C.

On the first day, a raw, uncooked eggplant sample was used as the control. All of the samples were macerated for a full day after the EO from the first set of samples and the *S. enterica* from the second group of tests were applied, gently mixed, and integrated. The samples were created using a CASO SV1000 sous vide device, produced by a company in Arnsberg, Germany. To prepare the samples for sous vide cooking, they were divided into groups and cooked at a specific temperature for a predetermined amount of time while being closely monitored.

The high-barrier polyethylene vacuum packaging bags are made of an impermeable substance that is resistant to moisture and extremely high or low temperatures (−30 °C to 100 °C), with a thickness between 40 and 200 microns. The data sheet also states that they taste and smell good, have a very long shelf life, and are free of bisphenol A and all plasticizers, including microplastics. They can last for several years when kept in freezers and refrigeration cases.

### 2.9. Microbiological Analyses of Eggplant Samples

Microbiological studies were carried out from day 0 to day 7. After heating, a portion of the samples were assessed one and seven days later. After weighing five grams, the eggplant samples were put in an aseptic stomacher bag. The materials were diluted to 10^−1^ in 45 mL of peptone water and then homogenized using a stomacher for two min. Subsequently, a standard pre-dried plate count agar medium was covered with 0.1 mL of an aliquot pipetted from an appropriate dilution. The samples were homogenized in the GFL 3031 shaking incubator (GFL, Burgwedel, Germany) for 30 min. The following microorganism populations were tested: Coliform bacteria were cultivated on Violet Red Bile Lactose Agar (VRBL, Oxoid, Basingstoke, UK) at 37 °C for 24 to 48 h and Total Viable Counts (TVCs) on Plate Count Agar (PCA, Oxoid, Basingstoke, UK) at 30 °C for 48 to 72 h [57].

### 2.10. Identification of Microbial Strains by MALDI-TOF MS Biotyper

The MALDI-TOF (Matrix-Assisted Laser Desorption/Ionization Time of Flight) MS Biotyper (Bruker, Daltonics, Bremen, Germany) and reference libraries were used for determining the eggplant samples. Once a stock solution had been prepared, it transformed into a substance that was organic. 50% acetonitrile, 47.5% water, and 2.5% trifluoroacetic acid made up the standard solution. 1 mL of stock solution was made by mixing 500 µL of pure 100% acetonitrile, 475 µL of filtered water, and 25 µL of pure 10% trifluoroacetic acid. In a 250 µL Eppendorf flask, the “HCCA matrix portioned” was created and homogenized with the organic solvent. The matrix materials were from Vrable, Slovakia-based Aloqence Science. The samples were created in accordance with previous suggestions [57].

### 2.11. Insect-Related Activity

The model organism utilized for assessing the insecticidal effectiveness of RDEO was *Megabruchidius dorsalis* Fahreus, 1839. Petri plates were used to hold groups of fifty *M. dorsalis* insects, and each plate was covered with sterile filter paper. To achieve concentrations of 100%, 50%, 25%, 12.5%, 6.25%, and 3.125%, RDEO was diluted with 0.1% polysorbate. After saturating sterile filter paper disks with 100 µL of each RDEO concentration, the plates were covered with parafilm and left at room temperature for an entire day. A 100 µL 0.1% polysorbate solution was given to the control group. After one full day, counts of live and dead insects were recorded. Three distinct studies successfully replicated this experimental setup [56].

### 2.12. Statistical Analysis

The data are presented as mean values ± standard deviation (SD), and each evaluation was carried out in triplicate. A one-way ANOVA was conducted, and then Tukey’s HSD test was run at *p* ≤ 0.05 of significance (CoStat version 6.451, CoHort Software, Pacific Grove, CA, USA). Finally, the JMP Pro 17.0 software program (SAS Institute, Cary, NC, USA) was used for the graphic elaborations.

## 3. Results

### 3.1. Chemical Composition of Rosa Damascena Essential Oil

The GC/MS technique was used to investigate the chemical composition of the RDEO volatile oil. The results, which show the percentage distribution of detected compounds, are presented in Table 1. A total of six volatiles were found, accounting for 99.9% of the EO composition. The findings suggest that the examined EO was primarily characterized by a significantly elevated percentage (70%) of phenylethyl alcohol.

### 3.2. Minimal Inhibition Concentration

The broth microdilution method was used to determine the MIC_50_ and MIC_90_, i.e., the minimum inhibitory concentrations. This was performed in order to gain a better understanding of the antibacterial activity of RDEO. Overall, RDEO had the strongest effect on inhibition of Gram-negative species. In particular, the lowest MIC_50_ values (0.250 mg/mL) and MIC_90_ values (0.13 and 0.14 mg/mL) were observed for *S. enterica* and *S. sonnei*. For Gram-positive *S. pneumoniae* and *S. aureus*, MIC_50_ (0.249 and 0.274 µL/mL, respectively) and MIC_90_ (0.283 and 0.291 mg/mL, respectively) were determined. The results of inhibiting *S. enterica* biofilm formation using RDEO showed MIBC_50_ values of 0.270 mg/mL and MIBC_90_ values of 0.291 mg/mL. The yeast strains *C. albicans* and *C. tropicalis* showed the best MIC results, with MIC_50_ and MIC_90_ values of 0.363 and 0.369 mg/mL and 0.393 and 0.386 mg/mL, respectively. The detailed results of the minimum inhibitory concentration (MIC) assay are presented in Table 2.

### 3.3. Antimicrobial Activity in Vapor Phase

**Figure 1:**
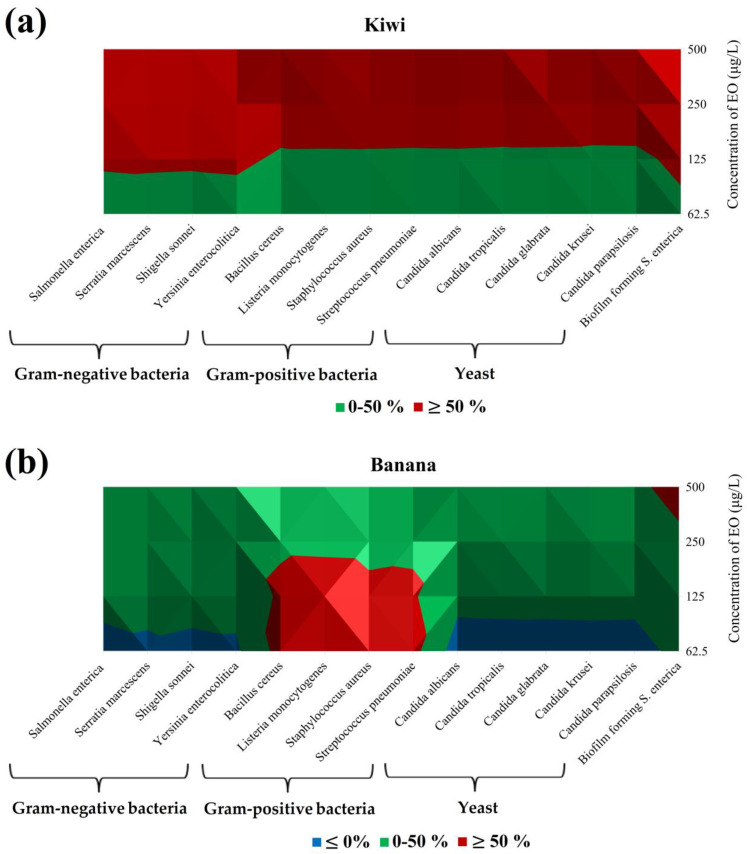
Isometric elaboration of the data of Table 4 (In situ analysis of the antimicrobial activity of the vapor phase of RDEO in fruits model): (**a**) Kiwi; (**b**) Banana. Blue ≤ 0%; Green: 0−50%; Red: ≥ 50%.

In the following experiment, we carried out an in situ antimicrobial investigation using fruit and vegetables as food models. The pre-selected bacteria have also been used in the in vitro evaluation. Table 3 and Figure 1a, b showed the vapor phase data for the fruit model. Overall, compared to the in vitro tests, the vapor phase of RDEO showed higher efficacy in suppressing Gram-positive strains, following yeasts and Gram-negative in the fruit model. When RDEO was used for in situ assessment of *C. tropicalis* development on the kiwi model, the results indicated that the highest concentration used (500 μg/L) had the strongest inhibitory effect (88.48%). The maximum dose of RDEO tested (88.32 and 87.55%, respectively) significantly inhibited the growth of *C. albicans* and *C. krusei* in the kiwi model (Table 3). RDEO was the most effective at inhibiting biofilm-forming *S. enterica* (76.36%) at 500 μg/L, but even at the highest concentration (500 μg/L), a significant level of inhibition was maintained compared to the other tested microorganisms. Furthermore, EO showed significant antibacterial activity against *S. pneumoniae* (86.89%). Maximum antibacterial activity against *S. sonnei* was observed at a concentration of 500 μg/L (76.46%). RDEO showed modest inhibitory effects in the in situ evaluation of Gram-positive bacterial development on the banana model. Among the Gram-positive strains, RDEO showed the highest inhibitory activity against *B. cereus* (67.13%) at 62.5 μg/L. In contrast, at the highest concentrations tested, RDEO showed the greatest inhibitory effect on biofilm-forming *S. enterica* (57.46%) among the Gram-negative strains growing on the banana model (Table 3). The results showed that among the yeasts grown in the banana model, RDEO, when applied at the maximum concentration, was the most effective at inhibiting the growth of *C. albicans* (46.83%) and *C. krusei* (45.63%). Furthermore, RDEO at the highest concentration tested strongly inhibited *Y. enterocolitica*. In the case of Gram-negative bacteria and yeasts, pro-microbial growth was detected in some of the species at the lowest concentrations, indicating that the concentrations of EO promoted the growth of the individual microorganisms.

**Figure 2:**
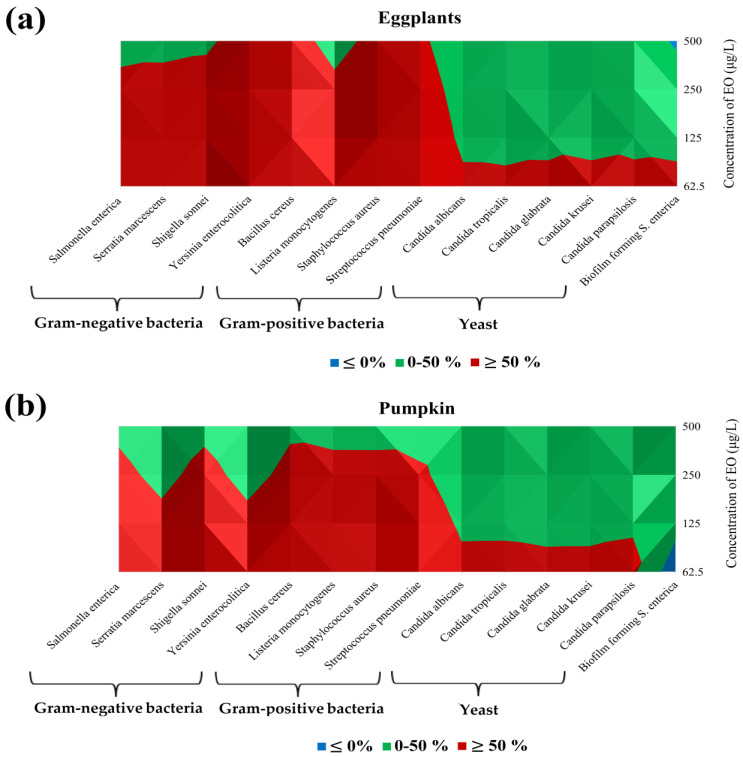
Isometric elaboration of the data of Table 5 (In situ analysis of the antimicrobial activity of the vapor phase of RDEO in vegetable models): (**a**) Eggplants; (**b**) Pumpkin. Blue ≤ 0%; Green: 0−50%; Red: ≥ 50%.

The results of the antibacterial activity on vegetables against all microorganisms observed in the vapor phase are reported in a subsequent section of our experiment (Table 4, Figure 2a,b). On the eggplant model, the best antimicrobial results were found against *S. pneumoniae* (85.62%) at the lowest concentration, followed by the bacteria *B. cereus* (85.13%), *S. aureus* (84.49%), and *L. monocytogenes* (82.41%), also at the lowest concentration of 62.5 μg/L (Table 4). Similarly, the lowest concentration of EO applied, 62.5 μg/L, showed the highest antimicrobial activity in the pumpkin model. The best results were obtained against *S. pneumoniae* (77.44%), followed by *S. enterica* with 76.62% inhibition and *L. monocytogenes* with 75.92% inhibition in the pumpkin model (Table 4). Satisfactory results were obtained against other Gram-positive and Gram-negative bacteria, while the lowest antimicrobial activity on the cucumber model was achieved by yeast against *C. parapsilosis* (58.62%) at the lowest RDEO concentration. In the case of the biofilm-forming bacterium *S. enterica*, pro-bacterial growth was detected at the lowest concentration.

In conclusion, as shown in Figure 1a,b, the two used fruit models (kiwi and banana), had different trends. In kiwi, by increasing the concentration of RDEO used, the % inhibition tends to increase. For all tested microorganisms, concentrations of 250 μg/L and 500 μg/L lead to a % inhibition greater than 50%; indeed, this inhibition value was maintained with 125 μg/L of RDEO for Gram-negative bacteria and BFB *S. enterica* (Figure 1a).

In banana, on the other hand, the 50% inhibition was archived only for Gram-positive bacteria for concentrations less than 125 μg/L and for BFB *S. enterica* at 500 μg/L. For the other microorganisms (such as yeast and Gram-negative bacteria), the RDEO had not shown a significant effect on inhibition. Indeed, the growth of microorganisms was favored at concentrations of 62.5 μg/L (Figure 1b).

On the contrary, in the vegetal models (eggplants and pumpkin) in Figure 2a,b, the similar trend has been seen. In fact, in this case, the inhibition percentage increased with decreasing the RDEO concentrations, indicating that a greater effect occurs at low concentrations (62.5 μg/L).

In eggplants, the application of RDEO showed the major effect. Gram-positive bacteria and Gram-negative bacteria were inhibited at concentrations lower than 250 μg/L, while for yeast and BFB *S. enterica* only at a concentration of 62.5 μg/L (Figure 2a).

In pumpkin, all Gram-positive bacteria and Gram-negative bacteria were inhibited at concentrations lower than 125 μg/L but at a concentration of 62.5 μg/L. Meanwhile, the RDEO promoted growth of BFB S. enterica (Figure 2b).

### 3.4. Antibiofilm Activity of Rosa Damascena

**Figure 3:**
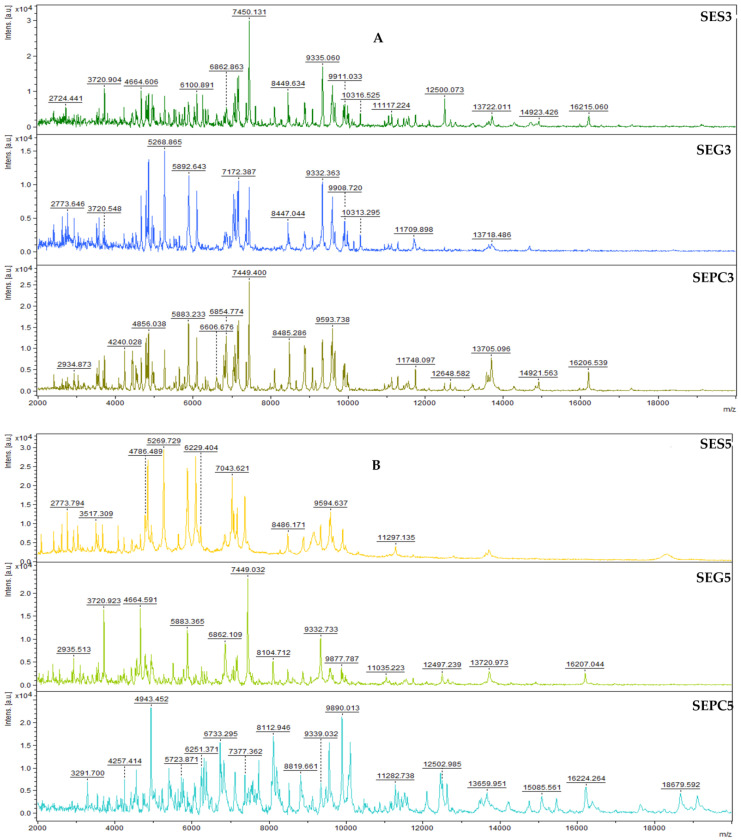

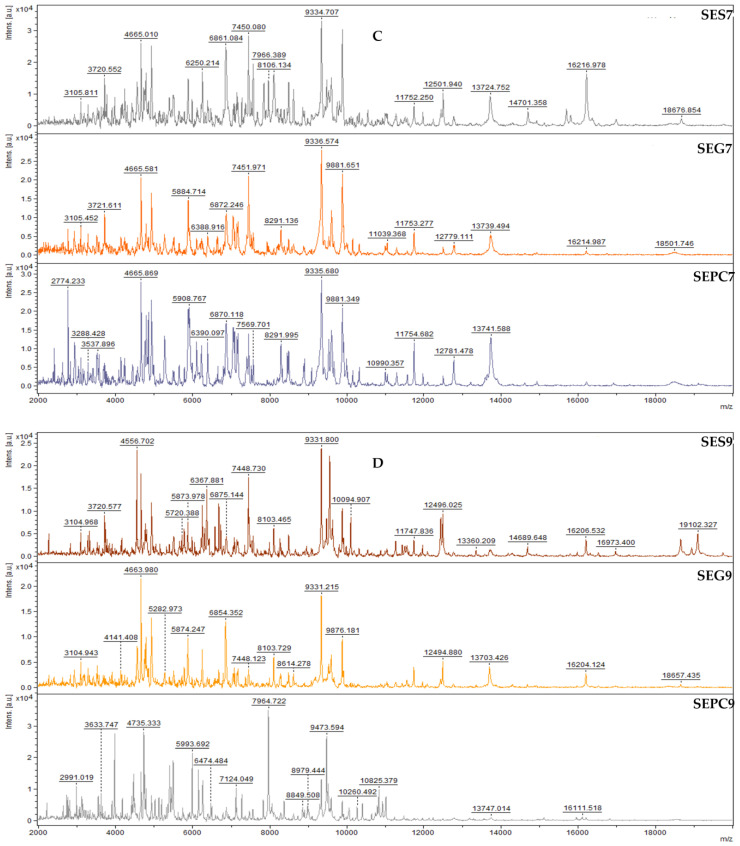

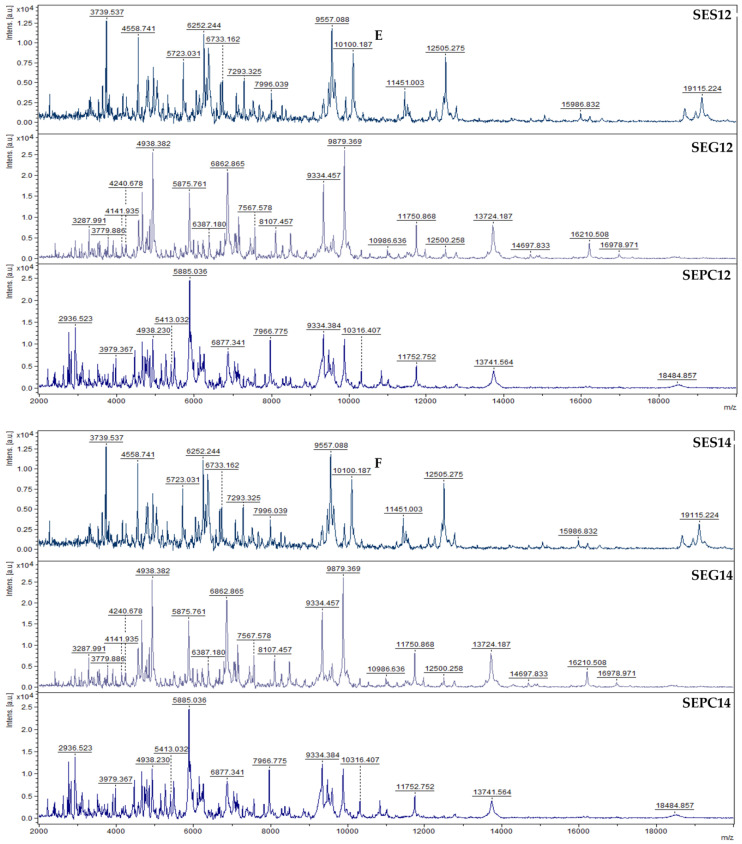
Representative MALDI-TOF mass spectra of *S. enterica*: (**A**) 3rd day; (**B**) 5th day; (**C**) 7th day; (**D**) 9th day; (**E**) 12th day; (**F**) 14th day. SE = *S. enterica*; G = glass; S = stainless steel; and PC = planktonic cells.

Figure 3A–F showed the spectra of the developmental stages of *S. enterica* biofilm throughout the experiment. RDEO was added to the experimental groups. The spectra were sorted into pairs according to their growth stage on different surfaces, except for the spectra of planktonic cells, which were obtained from the culture medium. Mass spectra of *S. enterica* on day 3 of the experiment are shown (Figure 3A). Spectra of control planktonic cells and experimental spectra with both stainless steel and glass are similar. On day 5 of the experiment (Figure 3B), a difference can be observed between the control planktonic cells and the experimental group with stainless steel, while the experimental group with glass is comparable. On day 7 of the experiment (Figure 3C), there was a change in the spectra in both experimental groups compared to the control planktonic cells. The same trend can be observed on day 9–14 of the experiment (Figure 3D,F).

**Figure 4:**
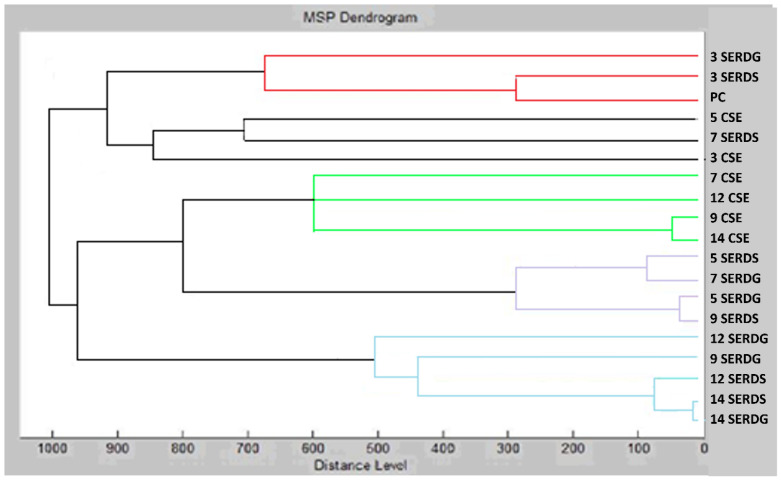
Dendrogram of *S. enterica* generated using MSPs of the planktonic cells and the control. SE = *S. enterica*; C = glass; S = stainless steel; and PC = planktonic cells.

The results suggest that distinct stages of biofilm development could be distinguished based on MSP distance using MALDI profiling. They were divided into different clusters (Figure 4). It can be observed that the planktonic stage of *S. enterica* showed the most significant similarity at day 3 with the experimental group in MSP distance. The control groups showed shorter MSP distances from the planktonic cells than the experimental groups on the following days.

### 3.5. Kinetic Growth of S. enterica

**Figure 5:**
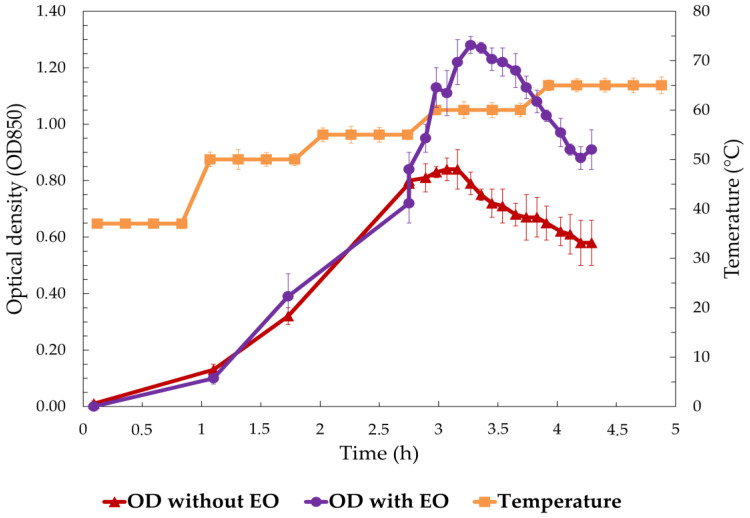
Optical density (OD850) of *S. enterica* over time with and without EO treatment at varying temperatures.

Figure 5 illustrates the growth of *S. enterica* over time, comparing the optical density (OD850) under two conditions: with 1% EO added at OD 1 (log phase) and without EO (control), alongside the temperature profile of the experiment. In the control group (without EO), *S. enterica* demonstrated continuous growth during the initial 3 h, reaching a peak OD of approximately 1.0. As the temperature increased beyond 40 °C after 3 h, bacterial growth stabilized, followed by a gradual decline in OD, indicating inhibited growth at higher temperatures. In contrast, the addition of 1% EO at the log phase resulted in a similar growth trend up to 3 h, with the OD peaking near 1.0. However, beyond the 3 h mark, when the temperature exceeded 40 °C, the OD began to decrease significantly in the EO-treated group. The decline in OD was more pronounced compared to the control, suggesting that the presence of EO enhanced the inhibitory effects of elevated temperature on bacterial growth. By the end of the experiment (4.5 h), the OD in the EO-treated group had dropped substantially lower than that of the control, indicating a marked reduction in bacterial viability. These findings highlight the combined effect of temperature and EO on bacterial inhibition.

**Figure 6:**
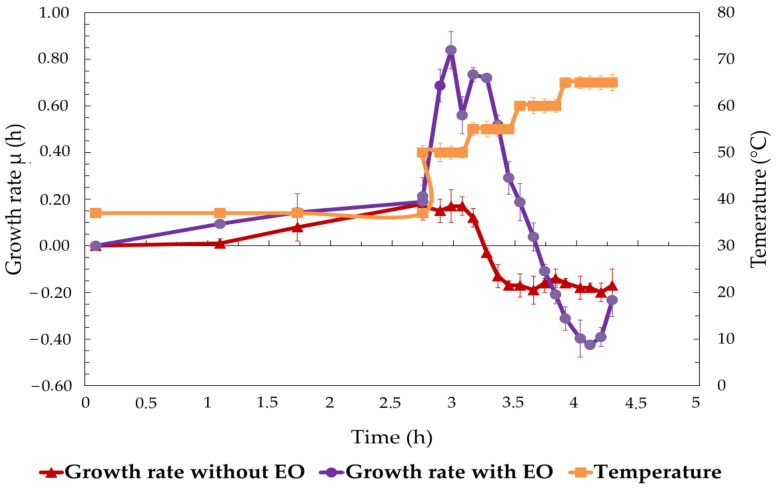
Growth rate (μ) of *S. enterica* over time with and without EO treatment at increasing temperatures.

Figure 6 depicts the growth rate (μ) of *S. enterica* over time, under two experimental conditions: with 1% EO added at the log phase (OD 1) and without EO (control), alongside the corresponding temperature profile. In the control condition (no EO), the bacterial growth rate increased steadily until approximately 3 h, when it began to plateau at approximately 0.2 h. After this point, the growth rate began to decrease, coinciding with an increase in temperature above 40 °C. At the end of the 4.5 h period, the growth rate dropped to negative values, indicating inhibition of the bacteria due to the elevated temperature. In the EO-treated conditions, the growth rate during the first 3 h had a similar pattern and peaked at approximately 0.4 h, slightly higher than the control group. However, after 3 h, the growth rate dropped dramatically and fell below the control as the temperature continued to increase. This sharp decrease in growth rate suggests a significant inhibitory effect of EO, especially when the temperature exceeded 40 °C. At the end of the experiment, the growth rate in the EO-treated group reached a more negative value compared to the control, indicating a stronger inhibition of bacterial growth.

These results demonstrate that EO in combination with increasing temperature significantly inhibits the growth of *S. enterica*.

### 3.6. Microbiological Quality of Sous Vide Eggplant

**Figure 7:**
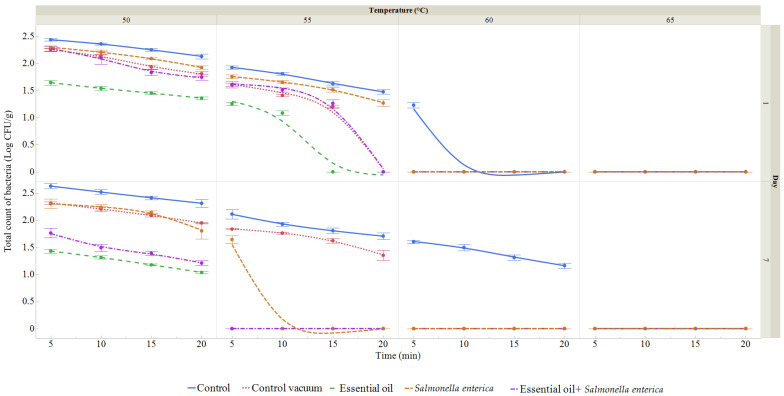
Total bacterial count (TBC) treated at temperatures ranging between 50 and 65 °C for durations of 5 to 20 min. (expressed in log CFU/g) on the first day and seventh day. Data are the mean (±SD) of three samples. Control: fresh samples were treated at 50–65 °C for 5 to 20 min after being packaged in polyethylene bags and kept at 4 °C. Control vacuum: fresh samples were treated at 50–65 °C for 5 to 20 min after being vacuum packed in polyethylene bags and kept at 4 °C. Ess. oil: vacuum-packed fresh samples were treated with 1% RDEO kept at 4 °C and treated for 5–25 min at 50–65 °C. *Salmonella*: vacuum-packed fresh samples were treated with *S. enterica* kept at 4 °C and treated for 5–20 min at 50–65 °C. *Salmonella* + EO: vacuum-packed fresh samples were treated with *S. enterica* and 1% RDEO was kept at 4 °C and treated for 5–20 min at 50–65 °C.

**Figure 8:**
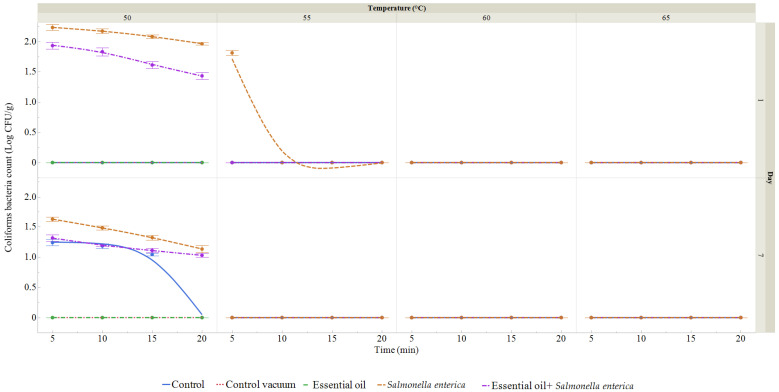
Coliform bacteria count (CBC) treated at temperatures ranging between 50 and 65 °C for durations of 5 to 20 min (expressed in log CFU/g) on the first and seventh day. Data are the mean (±SD) of three samples. Control: fresh samples were treated at 50–65 °C for 5 to 20 min after being packaged in polyethylene bags and kept at 4 °C. Control vacuum: fresh sample were treated at 50–65 °C for 5 to 20 min after being vacuum packed in polyethylene bags and kept at 4 °C. Ess. oil: vacuum-packed fresh samples were treated with 1% RDEO kept at 4 °C and treated for 5–25 min at 50–65 °C. *Salmonella*: vacuum-packed fresh sample treated with *S. enterica* was kept at 4 °C and treated for 5–20 min at 50–65 °C. *Salmonella* + EO: vacuum-packed fresh sample treated with *S. enterica* and 1% RDEO was kept at 4 °C and treated for 5–20 min at 50–65 °C.

A microbiological examination was conducted on raw eggplant to verify the presence of *Salmonella enterica* on XLD agar. On day 0, no coliforms were detected and the total bacterial count (TBC) was 1.98 log CFU/g. The TBC of the first day and the seventh day of storage were used to evaluate the microbiological quality of the vacuum-packed eggplant (Figure 7 and Figure 8). Within the control group, the range of the bacterial counts was from 1.18 to 2.45 log CFU/g and from 1.11 to 2.67 log CFU/g on the seventh day. TBC ranged from 1.19 to 2.26 log CFU/g on day 1 and 1.27 to 2.22 log CFU/g on day 7 in the vacuum-packed group. In the group that was vacuum packed and subjected to the RDEO treatment, the log CFU/g varied between 1.04 and 1.67 on the first day and between 1.06 and 1.43 on the seventh day. For TBC, the log CFU/g ranged from 1.22 to 2.32 on the first day and from 1.58 to 2.32 on the seventh day for the group treated with *S. enterica*. In the presence of *S. enterica*, TBC varied from 1.34 to 2.32 log CFU/g on day 1 and from 1.16 to 1.78 log CFU/g on day 7 after vacuum packaging and RDEO treatment. In general, there were fewer bacteria in the groups receiving RDEO and *S. enterica* inoculation.

Coliform bacteria count (CBC) was not identified in the RDEO group, in the vacuum-packed control group, or in the control group on the first day (Figure 8). Only the last two groups, where *S. enterica* was inoculated into the samples, revealed coliform bacteria on day 1. CBC ranged from 1.81 to 2.24 log CFU/g in the *S. enterica* inoculated group and from 1.37 to 1.95 log CFU/g in the RDEO with inoculation of *S. enterica* group. On the seventh day, the CBC in the control group ranged from 1.06 to 1.26 log CFU/g. Neither the RDEO group nor the vacuum-packed group contained coliforms after seven days of the experiment. In the group to which *S. enterica* was inoculated, the CBC ranged from 1.19 to 1.63 log CFU/g. In the treatment group receiving RDEO addition and inoculation with *S. enterica*, CBC ranged from 1.00 to 1.32 log CFU/g on day 7.

**Figure 9:**
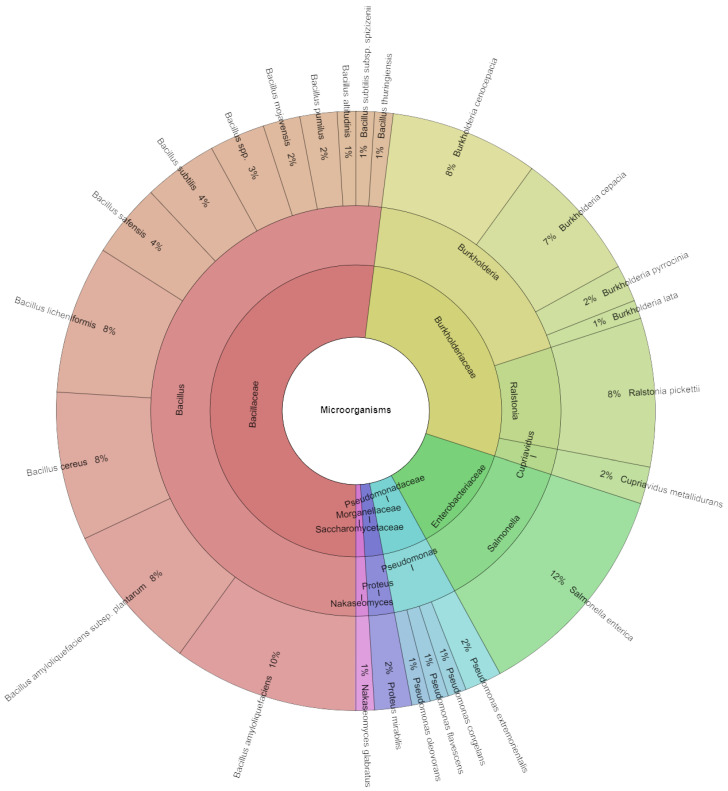
Krona chart: Species, genera, and families isolated from eggplant at the first day of storage.

The microbial species, genera, and families that have been isolated since the first day of storage are displayed in Figure 9. A total of 246 isolates were identified using mass spectrometry; the scores in all categories might go up to 2. There were twenty-five species, eight genera, and six families among these isolates. Most of the discovered species belonged to the families *Burkholderiaceae* and *Bacillaceae*. On the first day of the experiment, the most frequently recovered species from the sous vide eggplant samples in the experimental groups were *Salmonella enterica* (12%), *Bacillus amyloliquefaciens* (10%), *Bacillus cereus, Bacillus licheniformis, Burkholderia cenocepacia*, and *Ralstonia pickettii* (8%).

**Figure 10:**
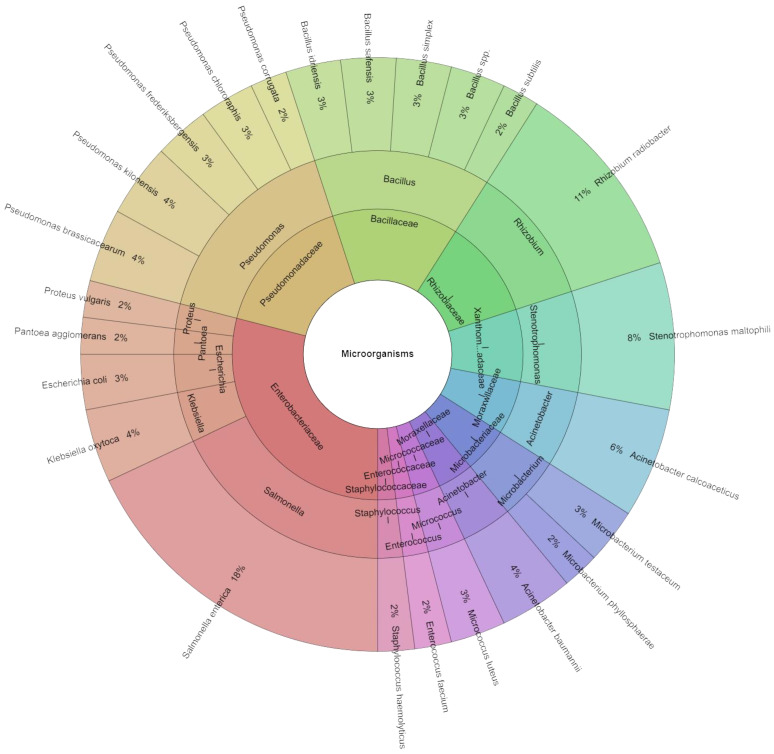
Krona chart: Species, genera, and families isolated from eggplant at the seventh day of storage.

The microbial species, genera, and families that were identified from sous vide-cooked samples of eggplant (*Solanum melongena*) after a seven-day period of preservation are displayed in Figure 10. A total of 363 isolates were found using mass spectrometry, and every group received a score of two or higher. In total, 24 species from 10 families and 14 genera were among these isolates. The Enterobacteriaceae and Pseudomonadaceae families contained the majority of the species. *S. enterica*, which was inoculated on eggplant, was the most commonly found isolate (18% of all isolates). After *S. enterica*, the most frequent bacteria were *Rhizobium radiobacter* (11%), *Stenotrophomonas maltophilia* (8%), *Acinetobacter calcoaceticus* (6%), *Acinetobacter baumannii, Klebsiella oxytoca,* and *Pseudomonas kilonensis*, each at 4%.

### 3.7. Insecticidal Activity of RDEO

The evaluation of the insecticidal activity of RDEO against *M. dorsalis* is presented in Table 5. According to the results, the application of 50% and 100% of the tested EO produced the highest levels of insecticidal activity. RDEO, when applied at a concentration of 6.25% and 3.125%, did not show any significant repellent effect against *M. dorsalis*. It is noteworthy that a concentration of 12.5% had an effect on the *M. dorsalis* population (50%). However, a concentration of 25% was effective against 70% of the insects, respectively.

## 4. Discussion

Terpenes, glycosides, flavonoids, and anthocyanins were among the components of *Rosa damascena* that were extracted from its flowers, petals, and hips (seed pods) [58,59,60]. Citronellol, geraniol, and nerol—the three main components of rose EO—have been shown to possess antibacterial properties [61,62]. Thus, these compounds may act as mediators for the antibacterial properties of the EO. The high concentration of phenylethyl alcohol in rose absolute may contribute to its antibacterial qualities, as alcohols have long been recognized for their antibacterial properties [63]. In our study, the main component was phenylethyl alcohol (70.0%), followed by citronellol (11.3%), geraniol (7.1%), nonadecane (4.7%), nerol (3.7%), and 1-nonadecene (3.1%). The obtained results agree with the studies by Ulusoy et al. [16] and Aydinli et al. [64] who showed that the main compound of rose absolute EO is characterized by the high relative amount of phenylethyl alcohol that varies in the range from 72.73% to 78.38%, followed by a significant abundance of citronellol, nonadecane, geraniol, and nerol. On the other hand, Lei et al. [65] showed that rose water from Chine had a higher amount of phenylethyl alcohol (90.2%). The principal constituents of rose EO extracted from the central region of Iran have been identified as citronellol, nonadecane, and geraniol [66]. According to other studies, the main constituents of the essential oil of *Rosa damascena* EO collected from northern Iran were 1-nonadecene, hexatriacontane, n-tricosane, and geraniol [67]. The most representative bioactive compounds of the essential oil of *Rosa damascena* EO collected from southern Iran were reported to be nonadecane, heneicosane, docosane, citronellol, and 9-nonadecene [67]. The principal constituents of the *Rosa damascena* EO extracted from the Kashan region were β-citronellol (14.88–47.43%), nonadecane (10.5–40.5%), geraniol (5.5–18%), and heneicosane (7–14%) [68].

The results showed that RDEO exhibited the strongest antibacterial action against Gram-positive *Streptococcus pneumoniae*, Gram-negative *S. sonnei*, and yeasts such as *Candida parapsilosis*. It has been demonstrated that RDEO exhibits antibacterial action against the biofilm-forming Gram-negative bacteria *Salmonella enterica*. The wide range of antibacterial activity of *Rosa damascena* has been established. Hydrosol, absolute, and EOs are significant products that demonstrate these benefits [62]. Strong antibacterial efficacy against strains of *Escherichia coli*, *Pseudomonas aeruginosa*, *Bacillus subtilis*, *Staphylococcus aureus*, *Chromobacterium violaceum*, and *Erwinia carotovora* was reported by Ulusoy et al. [16] using EO and absolute. *C. violaceum* was the microorganism most susceptible to rose absolute and EO. Additionally, *E. coli* was sensitive to rose essential oil. Nevertheless, none of the microbes were susceptible to the antibacterial effects of hydrosol [16]. Both Gram-positive and Gram-negative bacteria were also susceptible to the antibacterial properties of rose absolute. In a different investigation, the antibacterial properties of the EO extracted from the petals of *R. damascena* were assessed against three strains of *Xanthomonas axonopodis* spp. *vesicatoria*. The growth of the investigated strains of *X. axonopodis vesicatoria* was significantly reduced by the essential oil of *R. damascena* EO from flowers [69]. The antibacterial properties of *R. damascena* flowers were investigated against 15 different species of bacteria, including *Aeromonas hydrophila*, *Bacillus cereus*, *Enterobacter aerogenes*, *Enterococcus faecalis*, *E. coli*, *Klebsiella pneumoniae*, *Mycobacterium smegmatis*, *Proteus vulgaris*, *P. aeruginosa*, *P. fluorescens*, *Salmonella* Enteritidis, *Salmonella* Typhimurium, *S. aureus*, and *Yersinia enterocolitica* [70]. A different study demonstrated the in vitro antibacterial activity of the EO from *R. damascena* against *Pseudomonas*, *S. aureus*, and *E. coli*. In this work, *R. damascena* showed antibacterial efficacy against *S. aureus* [61]. The antibacterial activity of EOs from several plants, including *R. damascena*, was also evaluated against the yeast *Candida albicans*, Gram-positive *S. aureus*, Gram-negative *E. coli*, and Gram-negative *P. aeruginosa*. At low doses, the studied essential oils demonstrated both bactericidal and inhibitory effects against all tested bacteria [71]. In a different study, rose EO was found to be less effective against Gram-positive *S. aureus* compared to rifampin and gentamicin [72]. The same results were observed in our study. The antibacterial activity of the EO against this bacteria in Bulgaria and Saudi Arabia was greater than the lethal and inhibitory power of the EO against these bacteria [73]. Furthermore, in our work, we investigated the antimicrobial activity in the vapor phase on model fruits and vegetables. The results revealed that the inhibitory effect of RDEO at different concentrations acted on various microorganisms, depending on the model food. The best inhibitory effect was observed on kiwifruit against *C. tropicalis* yeast at the highest concentration, on banana against *B. cereus* at the lowest concentration, on eggplant against *S. pneumoniae*, and on pumpkin against *S. enterica* at the lowest concentrations. In some cases, pro-bacterial growth was demonstrated. A different study found antimicrobial effects in situ on fruit and vegetable models with similar results [54,55,56,57].

As far as we know, this is the first study to investigate the antibiofilm activity of RDEO. However, the current findings align with earlier research [74,75,76] that demonstrated the antibiofilm capabilities of various EOs against a range of foodborne pathogens on different surfaces. After employing RDEO to prevent the formation of *Salmonella enterica* biofilms, we obtained minimum inhibitory biofilm concentration (MIBC) values of 0.270 mg/mL and 0.291 mg/mL for MIBC_90_ using the crystal violet assay. The MALDI-TOF MS investigations indicated changes in the protein composition of the biofilm-forming bacteria *S. enterica*. The increasing incidence of bacterial diseases can be attributed to the lack of effective treatment options, antibiotic resistance, and the tendency of bacteria to form biofilms [77]. Consequently, new approaches that emphasize innovative technologies such as natural antimicrobials have emerged to control bacterial biofilms and mitigate the resistance of *Salmonella*, a highly prevalent pathogen. The EOs are antibacterial substances extracted from various herbal plants using a range of methods. EOs have been shown to possess significant antimicrobial activity against planktonic microbes, in addition to their antibacterial properties against microorganisms embedded in biofilm [78,79,80,81]. All examined free EOs and their active ingredients successfully inhibited planktonic bacterial cells [82,83]. However, the biofilms required significantly higher concentrations of Eos, or their active components, than their planktonic counterparts, to achieve comparable or zero microbial reductions [75,84,85,86,87,88,89]. In several studies, even when EOs or their active components were applied at twice or even higher concentrations than their minimum inhibitory concentrations (MIC) against planktonic cells, the biofilms were not completely eradicated [85,88,90,91,92,93,94,95,96]. Moreover, longer exposure times and greater EO concentrations were necessary to eliminate the biofilms that had formed over extended periods [80,97].

*Salmonella enterica* bacteria are responsible for a variety of severe, potentially fatal diseases in both humans and animals across the globe [98]. *Salmonella* grows best at 37 °C and pH 7, with a range of 5–47 °C and pH 4–9 [99]. In our study, without EO, the bacteria followed this growth pattern, thriving near 37 °C. However, with 1% EO, the growth was significantly inhibited, as shown by lower optical density (OD) and growth rates. At higher temperatures, both treated and untreated bacteria experienced a decline, but the EO-treated samples showed a steeper drop, highlighting EO’s stronger inhibitory effect even at suboptimal growth conditions.

The microbiological quality of eggplant was monitored during storage over 7 days. Eggplant was treated using the sous vide cooking method, while microbiological quality was assessed at different temperatures and times, in combination with *Salmonella enterica* inoculation and RDEO treatment. During the observation period, we found that increasing temperature and time positively affected the total number of microorganisms and the number of coliforms. RDEO treatment demonstrated an antimicrobial effect against *S. enterica* inoculation on eggplant. According to Zhou et al. [100], the shelf life of a green vegetable is determined by its ability to maintain an appealing appearance—a crisp, green texture with minimal browning or wetness—for consumers. After day 7 of storage, these products become more perishable than untreated materials due to off-flavors, tissue weakening, and microbial growth [101]. The relationship between the development of spoilage bacteria, their production of metabolites—particularly volatiles—and consumers’ is very important. Additionally, the inherent composition of food may affect bacterial susceptibility and reduce the antibacterial effectiveness of EOs. Higher concentrations of free EOs or their active ingredients are often required to achieve equivalent antimicrobial effects in food compared to in vitro assays [102,103,104,105]. This could have unfavorable organoleptic effects and reduce food products’ general appeal [106,107]. It was discovered that EOs or their active ingredients were more beneficial in foods with no or low fat content than in foods with high fat content [102,104,105]. Additionally, certain extrinsic factors like pH, temperature, and oxygen present may have an impact on the antibacterial action of EOs. Due to their higher hydrophobicity and easier dissolving in bacterial cell membranes, numerous EOs generally exhibited greater antibacterial action at low pH [102,103,108,109]. Some investigations found conflicting results regarding the effect of temperature on the antibacterial activity of EOs. Due to an increase in unsaturated phospholipids in the composition of cytoplasmic membranes and an increase in membrane fluidity, it has been discovered that lower temperatures (7 °C compared to 35 °C) boost the antibacterial action of the EOs or their active components [102]. According to Cava et al. [102], the enhanced fluidity makes the membrane connection weaker and facilitates the simpler dissolution of the EOs into it. In contrast, it was discovered that the antibacterial activity of carvacrol and cymene was more effective at 25 °C than it was at 15 and 4 °C [104]. The reason why bacterial cells were less sensitive to antimicrobial agents at lower temperatures remained unclear, but possible explanations included modifications to the fluidity and/or properties of the membrane, or the possibility that low temperatures would impact the synthesis of target sites in both EOs and bacterial cytoplasmic membranes, which in turn influences the susceptibility of microorganisms to EOs [104]. Additionally, EOs were reported to have greater antibacterial action in low-oxygen environments (vacuum and modified atmosphere packing) than in aerobic environments [110,111]. The microbiological quality of sous vide eggplant was associated with all the factors described above. That is, in our study, we investigated the effect of RDEO at 1% concentration as a function of temperature and time during storage for 7 days at 4 °C. In another study compared to our results, much higher numbers of microorganisms were obtained on eggplant treated with gamma irradiation and ascorbic acid [112]. On the first day of storage, the most frequently isolated species from sous vide eggplant were *Salmonella enterica, Bacillus amyloliquefaciens, Bacillus cereus, Bacillus licheniformis, Burkholderia cenocepacia*, and *Ralstonia pickettii*, and on the seventh day of storage they were *S. enterica, Rhizobium radiobacter, Stenotrophomonas maltophilia, Acinetobacter calcoaceticus, Acinetobacter baumannii, Klebsiella oxytoca,* and *Pseudomonas kilonensis*. In different study bacteria species namely, *Staphylococcus aureus, Bacillus subtilis, Micrococcus luteus*, and *Enterococcus faecalis,* and four fungal species, namely, *Saccharomyces, Fusarium, Aspergillus*, and *Rhizopus* were isolated from eggplant after preservative treatments [113].

The results indicated that applying 50% and 100% concentrations of the tested RDEO provided the highest insecticidal efficacy against *M. dorsalis*. This study represents the first investigation into the insecticidal activity of RDEO against *M. dorsalis*. Through a variety of mechanisms of action, such as contact activity, inhibition of molting and respiration, reduction in growth and fertility, cuticle destruction, and an impact on the octopamine pathway invertebrates, the EO exhibit a wide range of activity against insects and mites [114,115]. Geraniol and citronellol, the two main constituents of rose EO, shown contact actions against both adult and nymph *Tetranychus urticae*. On the adult and nymphal stages of *T. urticae*, the amounts of rose oil, geraniol, and citronellol that were applied, particularly after 96 h, had a strong fatal effect. At all concentrations, geraniol exhibited the greatest contact effect on *T. urticae* adults and nymphs, followed by citronellol and rose EO. According to Attia et al. [116], the primary constituents of the EO contribute to their acaricidal properties. EOs and monoterpenoids have been found to exhibit acaricidal efficacy against two-spotted mites [117,118,119,120,121]. The literature contains no reports on the insecticidal or acaricidal properties of rose EO. Nonetheless, research has been conducted on geraniol, an ingredient in several EOs.

## 5. Conclusions

The findings of this study confirm that *Rosa damascena* essential oil (RDEO) possesses potent antimicrobial and insecticidal properties, primarily attributed to its high concentration of phenylethyl alcohol. RDEO exhibited significant inhibitory effects against a variety of microorganisms, including clinically relevant Gram-positive and Gram-negative bacteria, as well as yeast strains. The antimicrobial activity was robustly demonstrated through minimum inhibitory concentration (MIC) assessments, indicating its effectiveness against biofilm-forming bacteria like *Salmonella enterica*. Additionally, the in situ analyses using fruit and vegetable models showcased the essential oil’s ability to inhibit microbial growth in vapor phase applications, highlighting its practical utility in food preservation. Furthermore, the study revealed effective antibiofilm activity, emphasizing the potential of RDEO to mitigate biofilm formation, a critical challenge in food safety and public health. The results also suggest that sous vide conditions may enhance the antimicrobial efficacy of RDEO, offering a novel approach to integrating EOs in cooking processes to improve food safety. Moreover, the insecticidal activity tests support the role of the EOs, as a natural pest deterrent, underscoring its applicability in sustainable agricultural practices. Given the rising concerns over antibiotic resistance, RDEO represents a promising candidate for further exploration as a safe and effective antimicrobial agent across various industries. Future studies should focus on elucidating the specific mechanisms of action of RDEO’s constituents and evaluating its effectiveness in real-world food systems and agricultural practices. Additionally, the research could investigate the formulation of RDEO with other natural preservatives to enhance its stability and efficacy and explore its potential synergistic effects in combination with conventional antimicrobial agents. By addressing these areas, RDEO could pave the way for innovative solutions to contemporary challenges in food safety and pest management.

## Figures and Tables

**Table 1:** Volatile constituents of RDEO.

No	RI (lit.) ^a^	RI (calc.) ^b^	Compound ^c^	% ^d^
1	1108	1115	phenylethyl alcohol	70.0
2	1229	1223	nerol	3.7
3	1225	1227	citronellol	11.3
4	1252	1249	geraniol	7.1
5	1875	1873	1-nonadecene	3.1
6	1990	1899	nonadecane	4.7
			Total	99.9

^a^ Literature values of retention indices on HP-5MS column; ^b^ Calculated values of retention indices on HP-5MS column; ^c^ identified compounds; ^d^ percentage amounts of identified compounds.

**Table 2:** Minimal inhibition concentration and minimal biofilm inhibition concentration of RDEO in mg/mL.

Microorganism	MIC_50_	MIC_90_
Gram-negative bacteria		
*Salmonella enterica* CCM 3807	0.250 ± 0.015 ^b^	0.277 ± 0.027 ^b^
*Serratia marcescens* CCM 8588	0.264 ± 0.014 ^b^	0.295 ± 0.006 ^b^
*Shigella sonnei* CCM 4421	0.250 ± 0.015 ^b^	0.282 ± 0.013 ^b^
*Yersinia enterocolitica* CCM 7204T	0.254 ± 0.015 ^b^	0.284 ± 0.010 ^b^
Gram-positive bacteria		
*Bacillus cereus* CCM 7934	0.352 ± 0.013 ^a^	0.382 ± 0.006 ^a^
*Listeria monocytogenes* CCM 4699	0.353 ± 0.029 ^a^	0.380 ± 0.003 ^a^
*Staphylococcus aureus* CCM 4423	0.274 ± 0.022 ^b^	0.291 ± 0.005 ^b^
*Streptococcus pneumoniae* CCM 4501	0.249 ± 0.029 ^b^	0.283 ± 0.007 ^b^
Yeast		
*Candida albicans* CCM 8186	0.363 ± 0.016 ^a^	0.393 ± 0.006 ^a^
*Candida tropicalis* CCM 8264	0.369 ± 0.012 ^a^	0.386 ± 0.011 ^a^
*Candida glabrata* CCM 8270	0.372 ± 0.014 ^a^	0.389 ± 0.011 ^a^
*Candida krusei* CCM 8271	0.369 ± 0.016 ^a^	0.389 ± 0.010 ^a^
*Candida parapsilosis* CCM 8260	0.372 ± 0.015 ^a^	0.387 ± 0.017 ^a^
Biofilm forming bacteria (BFB)	MIBC_50_	MIBC_90_
*Salmonella enterica*	0.270 ± 0.016 ^b^	0.291 ± 0.005 ^b^

Data are the mean (±SD) of three samples. Different letters in each column refer to significant differences (Tukey, *p ≤* 0.05).

**Table 3:** In situ analysis of the antimicrobial activity (%) of the vapor phase of RDEO in fruits model.

Food Model	Microorganisms	Concentration of RDEO in μg/L			
		62.5	125	250	500
Kiwi					
Gram-negative	*Salmonella enterica*	37.10 ± 2.28 ^b^	53.77 ± 1.06 ^a^	66.59 ± 1.72 ^a^	75.69 ± 1.05 ^b^
	*Serratia marcescens*	35.63 ± 1.76 ^b^	54.90 ± 1.68 ^a^	66.07 ± 2.24 ^a^	76.33 ± 2.19 ^b^
	*Shigella sonnei*	34.97 ± 2.53 ^b^	54.16 ± 0.82 ^a^	65.66 ± 2.21 ^a^	76.46 ± 2.10 ^b^
	*Yersinia enterocolitica*	37.13 ± 1.30 ^b^	55.25 ± 2.36 ^a^	66.09 ± 2.33 ^a^	76.15 ± 2.61 ^b^
Gram-positive	*Bacillus cereus*	25.22 ± 2.18 ^c^	46.29 ± 2.63 ^b^	64.36 ± 3.14 ^a^	85.50 ± 2.16 ^a^
	*Listeria monocytogenes*	25.62 ± 2.92 ^c^	46.13 ± 1.54 ^b^	66.68 ± 2.84 ^a^	86.56 ± 1.90 ^a^
	*Staphylococcus aureus*	25.40 ± 0.55 ^c^	46.33 ± 2.57 ^b^	65.97 ± 2.60 ^a^	85.05 ± 1.56 ^a^
	*Streptococcus pneumoniae*	25.69 ± 2.20 ^c^	45.88 ± 2.78 ^b^	66.06 ± 2.31 ^a^	86.89 ± 3.13 ^a^
Yeast	*Candida albicans*	24.41 ± 2.51 ^c^	46.14 ± 2.64 ^b^	66.76 ± 2.92 ^a^	88.32 ± 1.15 ^a^
	*Candida tropicalis*	25.43 ± 2.14 ^c^	45.48 ± 1.61 ^b^	65.69 ± 2.94 ^a^	88.48 ± 1.14 ^a^
	*Candida glabrata*	24.80 ± 1.16 ^c^	45.29 ± 1.91 ^b^	67.06 ± 2.41 ^a^	84.93 ± 1.70 ^a^
	*Candida krusei*	25.70 ± 1.88 ^c^	45.00 ± 2.19 ^b^	64.85 ± 3.18 ^a^	87.55 ± 2.80 ^a^
	*Candida parapsilosis*	26.09 ± 3.05 ^c^	45.21 ± 2.32 ^b^	65.15 ± 0.51 ^a^	86.37 ± 2.94 ^a^
BFB	*Salmonella enterica*	44.26 ± 1.65 ^a^	55.30 ± 1.84 ^a^	66.43 ± 1.70 ^a^	76.38 ± 3.51 ^b^
Banana					
Gram-negative	*Salmonella enterica*	−14.77 ± 2.11 ^de^	13.63 ± 1.16 ^d^	26.06 ± 1.68 ^e^	36.44 ± 1.70 ^cd^
	*Serratia marcescens*	−6.74 ± 0.97 ^c^	10.73 ± 0.77 ^d^	24.40 ± 2.09 ^e^	36.84 ± 2.21 ^cd^
	*Shigella sonnei*	−12.77 ± 3.90 ^cd^	17.50 ± 1.67 ^d^	26.76 ± 2.91 ^de^	36.40 ± 2.30 ^cd^
	*Yersinia enterocolitica*	−7.85 ± 1.08 ^cd^	16.33 ± 2.57 ^d^	35.73 ± 2.86 ^c^	42.10 ± 1.40 ^bc^
Gram-positive	*Bacillus cereus*	67.13 ± 2.29 ^a^	56.68 ± 1.21 ^ab^	45.47 ± 3.60 ^a^	35.76 ± 1.83 ^cd^
	*Listeria monocytogenes*	65.76 ± 4.21 ^a^	63.52 ± 2.11 ^a^	44.67 ± 1.06 ^a^	36.03 ± 1.68 ^cd^
	*Staphylococcus aureus*	66.08 ± 2.33 ^a^	55.43 ± 2.30 ^b^	44.08 ± 1.38 ^ab^	33.63 ± 0.96 ^d^
	*Streptococcus pneumoniae*	65.37 ± 2.28 ^a^	54.33 ± 1.39 ^b^	45.61 ± 2.31 ^a^	35.66 ± 2.21 ^cd^
Yeast	*Candida albicans*	−23.37 ± 2.96 ^f^	14.60 ± 2.17 ^d^	36.77 ± 2.20 ^bc^	46.83 ± 1.86 ^b^
	*Candida tropicalis*	−22.40 ± 1.10 ^ef^	15.70 ± 2.95 ^d^	36.81 ± 1.85 ^bc^	45.43 ± 4.35 ^b^
	*Candida glabrata*	−23.33 ± 1.40 ^f^	16.39 ± 3.55 ^d^	33.62 ± 2.72 ^cd^	44.74 ± 2.63 ^b^
	*Candida krusei*	−22.48 ± 4.12 ^ef^	17.56 ± 3.96 ^d^	37.11 ± 2.27 ^bc^	45.63 ± 2.63 ^b^
	*Candida parapsilosis*	−22.93 ± 1.00 ^f^	16.39 ± 3.55 ^d^	34.19 ± 3.38 ^cd^	45.61 ± 2.32 ^b^
BFB	*Salmonella enterica*	15.66 ± 3.32 ^b^	35.39 ± 3.22 ^c^	45.66 ± 3.32 ^a^	57.46 ± 3.01 ^a^

Data are the mean (± SD) of three samples. Different letters in each column (for each fruit type: kiwi and banana) refer to significant differences (Tukey, *p ≤* 0.05).

**Table 4:** In situ analysis of the antimicrobial activity (%) of the vapor phase of RDEO in vegetable models.

Food Model	Microorganisms	Concentration of RDEO in μg/L			
		62.5	125	250	500
Eggplants					
Gram-negative	*Salmonella enterica*	76.34 ± 2.11 ^bc^	65.20 ± 3.28 ^c^	56.29 ± 2.46 ^bc^	42.59 ± 1.94 ^b^
	*Serratia marcescens*	76.03 ± 2.78 ^bc^	65.87 ± 4.44 ^bc^	56.09 ± 4.14 ^bc^	45.03 ± 1.68 ^b^
	*Shigella sonnei*	74.43 ± 3.49 ^c^	66.08 ± 1.21 ^bc^	56.86 ± 1.76 ^bc^	47.19 ± 0.61 ^b^
	*Yersinia enterocolitica*	86.29 ± 4.29 ^a^	74.19 ± 0.64 ^a^	63.15 ± 1.58 ^ab^	57.79 ± 1.75 ^a^
Gram-positive	*Bacillus cereus*	85.15 ± 0.51 ^a^	75.93 ± 4.32 ^a^	63.30 ± 2.24 ^ab^	57.51 ± 1.69 ^a^
	*Listeria monocytogenes*	82.41 ± 1.11 ^ab^	63.37 ± 2.25 ^c^	54.41 ± 3.81 ^c^	43.52 ± 1.89 ^b^
	*Staphylococcus aureus*	84.49 ± 1.86 ^a^	73.75 ± 2.17 ^ab^	64.76 ± 3.50 ^a^	55.74 ± 0.93 ^a^
	*Streptococcus pneumoniae*	85.62 ± 2.26 ^a^	74.58 ± 3.93 ^a^	67.87 ± 0.02 ^a^	57.17 ± 1.22 ^a^
Yeast	*Candida albicans*	56.14 ± 1.18 ^d^	43.89 ± 0.58 ^d^	35.70 ± 1.88 ^d^	24.33 ± 2.26 ^c^
	*Candida tropicalis*	54.74 ± 2.71 ^d^	43.67 ± 2.06 ^d^	35.37 ± 1.59 ^d^	25.69 ± 3.53 ^c^
	*Candida glabrata*	54.56 ± 2.01 ^d^	46.03 ± 2.56 ^d^	33.63 ± 1.11 ^d^	23.96 ± 3.40 ^c^
	*Candida krusei*	57.49 ± 0.62 ^d^	43.47 ± 2.36 ^d^	34.97 ± 3.41 ^d^	25.33 ± 2.28 ^c^
	*Candida parapsilosis*	54.86 ± 1.72 ^d^	46.06 ± 2.23 ^d^	35.66 ± 2.72 ^d^	25.74 ± 2.10 ^c^
BFB	*Salmonella enterica*	56.03 ± 2.31 ^d^	44.20 ± 1.29 ^d^	24.90 ± 2.73 ^e^	−6.40 ± 1.65 ^d^
Pumpkin					
Gram-negative	*Salmonella enterica*	76.62 ± 4.15 ^a^	66.54 ± 1.07 ^a^	55.66 ± 2.93 ^a^	45.59 ± 2.33 ^a^
	*Serratia marcescens*	65.28 ± 3.75 ^bc^	55.88 ± 2.59 ^bc^	44.34 ± 1.25 ^b^	35.10 ± 1.68 ^b^
	*Shigella sonnei*	72.96 ± 1.79 ^ab^	64.37 ± 1.72 ^ab^	56.81 ± 1.05 ^a^	45.00 ± 1.25 ^a^
	*Yersinia enterocolitica*	73.59 ± 4.31 ^ab^	55.84 ± 4.51 ^bc^	43.37 ± 2.07 ^b^	34.00 ± 3.69 ^bc^
Gram-positive	*Bacillus cereus*	75.15 ± 1.57 ^a^	65.88 ± 4.05 ^a^	55.34 ± 1.71 ^a^	46.86 ± 1.14 ^a^
	*Listeria monocytogenes*	75.92 ± 3.98 ^a^	64.59 ± 1.97 ^ab^	56.14 ± 3.71 ^a^	44.12 ± 2.94 ^a^
	*Staphylococcus aureus*	74.38 ± 3.17 ^a^	62.95 ± 8.21 ^ab^	56.06 ± 2.78 ^a^	44.06 ± 0.55 ^a^
	*Streptococcus pneumoniae*	77.44 ± 1.67 ^a^	65.92 ± 0.58 ^a^	56.50 ± 1.19 ^a^	33.96 ± 0.71 ^bc^
Yeast	*Candida albicans*	56.14 ± 1.19 ^d^	46.32 ± 2.10 ^cd^	32.66 ± 2.00 ^cd^	24.80 ± 3.61 ^d^
	*Candida tropicalis*	56.44 ± 1.63 ^cd^	46.41 ± 0.64 ^cd^	34.63 ± 1.00 ^c^	27.42 ± 2.18 ^cd^
	*Candida glabrata*	55.63 ± 1.23 ^d^	44.70 ± 0.96 ^d^	33.40 ± 1.45 ^cd^	23.56 ± 2.11 ^d^
	*Candida krusei*	55.94 ± 2.79 ^d^	44.73 ± 3.81 ^d^	35.06 ± 3.27 ^c^	27.88 ± 1.10 ^cd^
	*Candida parapsilosis*	58.60 ± 0.66 ^cd^	46.38 ± 2.76 ^cd^	35.29 ± 3.21 ^c^	25.30 ± 3.36 ^d^
BFB	*Salmonella enterica*	−29.52 ± 5.26 ^e^	16.03 ± 1.68 ^e^	26.77 ± 2.93 ^d^	34.15 ± 3.43 ^bc^

Data are the mean (±SD) of three samples. Different letters in each column (for each vegetable type: eggplants and pumpkin) refer to significant differences (Tukey, *p ≤* 0.05).

**Table 5:** Insecticidal activity of RDEO against *Megabruchidius dorsalis* (n = 50).

Concentration (%)	Number of Living Individuals	Number of Dead Individuals	Insecticidal Activity (%)
100	10	90	90.00 ± 0.00
50	20	80	80.00 ± 0.00
25	30	70	70.00 ± 0.00
12.5	50	50	50.00 ± 0.00
6.25	100	0	0.00 ± 0.00
3.125	100	0	0.00 ± 0.00
Control group	100	0	0.00 ± 0.00

## Data Availability

The original contributions presented in the study are included in the article, further inquiries can be directed to the corresponding author.
